# The Role of the Oral Immune System in Oropharyngeal Candidiasis-Facilitated Invasion and Dissemination of *Staphylococcus aureus*

**DOI:** 10.3389/froh.2022.851786

**Published:** 2022-04-07

**Authors:** Raymond Pasman, Bastiaan P. Krom, Sebastian A. J. Zaat, Stanley Brul

**Affiliations:** ^1^Department of Molecular Biology and Microbial Food Safety, Swammerdam Institute for Life Sciences, University of Amsterdam, Amsterdam, Netherlands; ^2^Department of Preventive Dentistry, Academic Centre for Dentistry Amsterdam (ACTA), University of Amsterdam and Vrije Universiteit Amsterdam, Amsterdam, Netherlands; ^3^Department of Medical Microbiology and Infection Prevention, Amsterdam UMC, University of Amsterdam, Amsterdam Institute for Infection and Immunity, Amsterdam, Netherlands

**Keywords:** bloodstream infection (BSI), *Candida albicans*, *Staphylococcus aureus*, oral, immunocompromised

## Abstract

*Candida albicans* and *Staphylococcus aureus* account for most invasive fungal and bacterial bloodstream infections (BSIs), respectively. However, the initial point of invasion responsible for *S. aureus* BSIs is often unclear. Recently, *C. albicans* has been proposed to mediate *S. aureus* invasion of immunocompromised hosts during co-colonization of oral mucosal surfaces. The status of the oral immune system crucially contributes to this process in two distinct ways: firstly, by allowing invasive *C. albicans* growth during dysfunction of extra-epithelial immunity, and secondly following invasion by some remaining function of intra-epithelial immunity. Immunocompromised individuals at risk of developing invasive oral *C. albicans* infections could, therefore, also be at risk of contracting concordant *S. aureus* BSIs. Considering the crucial contribution of both oral immune function and dysfunction, the aim of this review is to provide an overview of relevant aspects of intra and extra-epithelial oral immunity and discuss predominant immune deficiencies expected to facilitate *C. albicans* induced *S. aureus* BSIs.

## Introduction

Various niches of the healthy human body are readily colonized by commensal fungi and bacteria. However, numerous of these microbes are opportunistic pathogens, *i.e*. they become pathogenic when the environment allows it. One major opportunistic pathogen colonizing the oral cavity, gastro- intestinal tract, and vagina of most healthy individuals is *Candida albicans* [1]. *C. albicans* is a polymorphic fungus able to grow as relatively harmless yeast and pseudohyphal cells, as well as harmful invasive hyphae [2]. Immunocompromised individuals suffering from suppressed extra epithelial oral immunity are prone to develop oropharyngeal candidiasis (OPC), a local infection of oral mucosa characterized by epithelium invading hyphae. If intra-epithelial immune responses are unable to prevent further growth, invading cells can disseminate and result in life threatening blood stream infections (candidemia) [3, 4]. Candidemia is linked to severe morbidity and mortality, with the latter reaching up to 71% depending on patient age and/or underlying conditions [5]. Approximately one in five candidemia cases is known to be polymicrobial [6, 7], with *Staphylococcus aureus*, a leading cause of bloodstream infections (BSIs) [7, 8], as one of the most common co-isolated bacterial species [6]. Interestingly, a significant number of patients suffering from staphylococcal BSIs have no reported porte d'entrée [9]. Recent findings suggest that *C. albicans* invasion of the oral epithelium creates such a porte d'entrée and, thereby, facilitates *S. aureus* BSIs [10–13]. This process was first hypothesized to be facilitated by hyphae adhering *S. aureus* moving along with the growing hyphae in a hitchhiking like manner [14]. However, recent research has shown *S. aureus* to remain situated at the initial point of adhesion during hyphal growth, rendering the co-invasion hypothesis up for debate [11]. Even though the specific mechanisms driving co-invasion and dissemination remain to be determined, it is apparent that it majorly depends on hyphal invasion (both mechanically and with aid of the secreted cytotoxic peptide candidalysin), and the binding of *S. aureus* to the hyphal agglutinin like sequence 1 and 3 (Als1 and Als3) proteins [11–14]. Importantly, several new lines of evidence also point to a crucial role of the oral immune system in this process [10, 13]. Whereas low level immune suppression is crucial for OPC development and *S. aureus* co-colonization in murine models, severe immune suppression significantly reduces *S. aureus* dissemination [13, 15]. Thus far, this reduction has been attributed to a significant reduction in local phagocyte numbers [13]. When present, phagocytes are actively recruited by *C. albicans* hyphae, but are unable to engulf them and internalize hyphae bound *S. aureus* instead [11]. *S. aureus* is notorious for circumventing phagocytic killing and could, thus, utilize phagocytes as a trojan horse while it is transported to draining cervical lymph nodes, facilitating further dissemination to the bloodstream [11, 16]. Thereby, oral immune dysfunction could induce OPC facilitated *S. aureus* BSIs without instigating candidemia and also account for monomicrobial *S. aureus* BSIs. Considering the crucial contribution of immune dysfunction, immunocompromised individuals might not only be at increased risk of developing OPC but *S. aureus* BSIs as well. Due to the fact that immunosuppression affects approximately one in every 16 individuals and is increasing with time, its effect on OPC induced *S. aureus* BSIs can be more prominent than anticipated [17]. In light of importance of the oral immune system in this process, the aim of this review is to provide a detailed overview of both extra-epithelial and intra-epithelial interactions between the oral immune system and *C. albicans* and *S. aureus*. Furthermore, predominant immunosuppressive disorders linked to these interactions and their relation to increased risk of OPC induced *S. aureus* dissemination will be discussed.

## Extra-Epithelial Oral Immunity

Extra-epithelial oral immunity encompasses immune factors present/secreted in saliva and gingival crevicular fluid (GCF). These immune factors include antimicrobial proteins and antimicrobial peptides (AMPs), oral polymorphonuclear leukocytes and factors of the complement system. Extra-epithelial oral immunity is continuously active to control commensal colonization and prevent pathogenic (over)growth (*e.g*. of *C. albicans*). Below all relevant aspects of extra-epithelial oral immunity will be discussed in relation to their general role in the oral cavity as well as their role in *C. albicans* and *S. aureus* immunity. Possible mechanisms of both organisms to evade extra-epithelial oral immunity will be covered as well.

### Antimicrobial Proteins and Peptides

**Figure 1:**
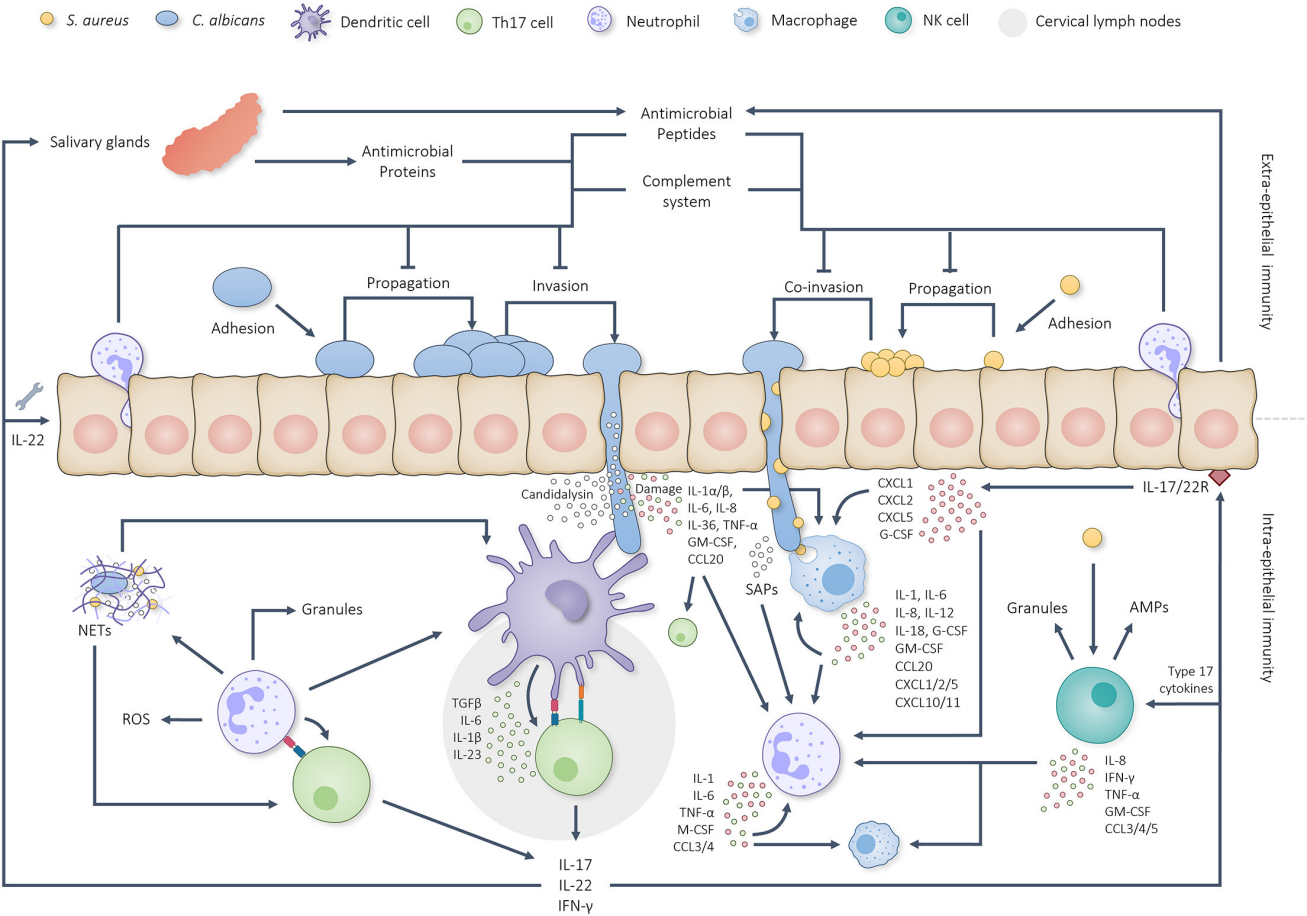
Graphical overview of the intricate interplay between the oral immune system and *C. albicans/S. aureus* infections. *C. albicans* first adheres to the oral epithelium, starts propagating and initiates hyphal growth. Extra-epithelial antimicrobial proteins, AMPs, complement factors and neutrophils limit pathogenic overgrowth and tissue invasion. Hyphal invasion, induced tissue damage and candidalysin induce a cascade of intra-epithelial immune reactions. Dendritic cells are able to take up and present pathogenic antigens to naïve T cells in the cervical lymph nodes which, together with IL-1, IL-6 and IL-23, stimulate Th17 differentiation. Simultaneously, oral EC produce IL-1α, IL-1β and IL-36 to activate other type 17 cells. Together, the activated type 17 cells start producing IL-17, IL-22 and IFN-γ. IL-17 and IL-22 sequentially trigger the corresponding receptors on oral ECs and stimulate the production/secretion of both AMPs (also by salivary glands) and chemokines plus aid in the repair of damaged barrier areas. Secreted chemokines attract more neutrophils and macrophages to the site of infection and stimulate their activation. Neutrophils and macrophages phagocytose and break down *C. albicans* yeast cells, hyphal fragments and *S. aureus* cells besides which they produce various cytokines and chemokines to further stimulate phagocyte attraction and activation. Phagocyte attraction and activation is also stimulated by candidal and staphylococcal activated NK cells. Neutrophils respond to activation by phagocytosing and killing the threat when possible, producing NETs, secreting granular components and utilizing ROS. Additionally, neutrophils have been found able to prime/activate T cells and APCs besides which they are able to produce extra cytokines and chemokines to stimulate Th17 differentiation and further neutrophil attraction/activation. This positive feedback loop continues until the microbial threat has been eliminated.

Inside the oral cavity cells of the epithelium and salivary glands continuously produce and secrete antimicrobial proteins and AMPs into saliva and GCF (Figure 1) [18–22]. Predominant oral antimicrobial proteins include lysozyme, lactoferrin, and lactoperoxidase and reduce microbial growth by breaking down peptidoglycan residues, sequestering iron, and oxidating various microbial substrates, respectively. Oral AMPs include α-defensins, β-defensins, LL-37, and histatins and likely exert their antimicrobial efficacy through insertion into cell membranes, lethally destabilizing the membrane [23]. In addition to their direct antimicrobial effect, AMPs serve as chemoattractants for immature dendritic cells, neutrophils, monocytes, and various T-cells and induce the secretion of pro-inflammatory cytokines and chemokines [24, 25]. While low levels of AMPs are constitutively expressed and secreted they can be strongly upregulated following the activation of pattern recognition receptors (PRRs) by specific microbial pathogen-associated molecular patterns (PAMPs) [26–29].

Oral epithelial cells (ECs) and cells of the innate immune system utilize PRRs such as Toll-like receptors (TLR), Nucleotide-Binding Oligomerization Domain Receptors (NODs), Protease-Activated Receptors (PARs), C-type lectin receptors (CLR) and RIG-1-like receptors (RLR) to distinguish *C. albicans* yeast from hyphal PAMPs and adapt their immune response accordingly [30]. Regarding human oral ECs all TLRs have been found present with TLR5 and 7, however, lacking on the surface of buccal epithelial cells [31]. Of these TLRs, TLR2, 4 and 6 have been found to directly affect the mucosal defense against *C. albicans* through binding of cell wall mannans (TLR2/4/6) and chitin (TLR4) [27, 31]. Additionally, the ephrin type-A receptor 2 (EphA2), has been identified as a non-traditional epithelial PRR able to recognize *C. albicans* β-glucans [32]. The distinction between yeast and hyphae is mainly accomplished through binding of EC receptor tyrosine kinases (RTKs) such as the human epidermal growth factor receptor 2 (Her2) and EGF, to the hyphal Als3 and Ssa1 proteins [33]. Recently, EGFR has also been found to constitutively associate with the EphA2 receptor to form a physical complex that, in response to Als3, sustains EphA2 activation [34]. Whereas the cell wall composition of *C. albicans* yeast cells provokes only a weak activation of AMP release, cell wall components of hyphae trigger a more strong and sustained secretion [29, 30]. In response, *C. albicans* utilizes three mechanisms to reduce AMP efficacy: (1) secretion of proteins able to bind and/or degrade AMPs, (2) extrusion of internalized AMPs via efflux transporters, and (3) downregulation of cellular stress response pathways resulting in cell wall adaptations and both reduced ROS production and ATP efflux crucial for AMP killing [35]. Besides protecting *C. albicans* cells, secreted AMP inhibiting/degrading proteins are known to provide protection to concurrently present bacteria such as *S. aureus* [36]. Additional to AMP induction, PAMPs of *C. albicans* hyphae and produced candidalysin activate the epidermal growth factor receptor (EGFR) and ephrin type-A receptor 2 (EphA2) of oral ECs and, thereby, induce the release of pro-inflammatory cytokines and chemokines which attract neutrophils to the site of infection [37, 38].

Concerning *S. aureus*, when reaching sufficient biomass and virulence, various PAMPs are able to activate PRRs and promote AMP secretion as well [39–43]. Even though specific research on oral EC recognition of *S. aureus* is still lacking, general recognition of *S. aureus* occurs via PRRs, including TLRs (especially TLR2), NOD-2 and TNF-α receptor 1 (TNFR1) which bind staphylococcal specific PAMPs such as cell surface protein A, peptidoglycan, LTA, phenol-soluble modulins (PSMs) and hemolysins [44]. Numerous strains of *S. aureus* are, however, able to utilize sensor/regulator systems to sense AMPs and adapt their transcriptomic profile accordingly [45]. These transcriptomic shifts can result in the insertion of positively charged D-alanine and L-lysine into the highly negatively charged cell wall lipoteichoic acid and phosphatidylglycerol, respectively, both reducing the affinity of cationic AMPs [45–48]. AMP efficacy can also be directly suppressed by *S. aureus* via the secretion of the α-defensins inhibiting staphylokinase and LL-37 degrading aureolysin [46, 47]. Whereas *C. albicans* is known to induce oral epithelial cytokine production, *S. aureus* has not yet been studied in this context. However, *S. aureus* is known to induce the secretion of pro-inflammatory cytokines and chemokines in nasal, corneal, and vaginal ECs, rendering it likely that oral ECs could act similarly [49].

A final class of proteins able to prevent extra epithelial oral microbial pathogenesis is immunoglobulins [50]. A key mechanism in prevention of pathogenesis is immune exclusion, primarily facilitated by secretory immunoglobulin A (SIgA), the major immunoglobulin in saliva [51]. Immune exclusion encompasses antibody coating of microbes which limits epithelial contact and invasion of pathogens and antigens [51]. However, whereas SIgA is indeed able to reduce the adherence of *C. albicans* to human epithelial cells [52], other factors present in saliva, such as secretory component (SC), actually promote adhesion beyond the inhibitory effects of SIgA, rendering immune exclusion of *C. albicans* unlikely [52, 53]. Even though studies regarding the effects of *S. aureus* and SIgA are limited, SIgA has been found to abundantly bind to the surface of *S. aureus* but, however, binding is reduced during biofilm growth [54]. When SIgA is immobilized onto polyvinyl microtiter plates, adhesion of *S. aureus* is not promoted, suggesting SIgA reduces *S. aureus* adhesion to at least solid surfaces [55]. Since *C. albicans* can be considered a solid surface for colonization of *S. aureus* SIgA could impact this interaction. It should be noted that the effect of this induced immune deficiency on SIgA has not yet been reported [10, 11, 13]. Therefore, the exact role of SIgA in the immune exclusion of *S. aureus* and the possibility of adhesion promoting salivary proteins and *C. albicans* remains to be determined.

### Neutrophils

**Figure 2:**
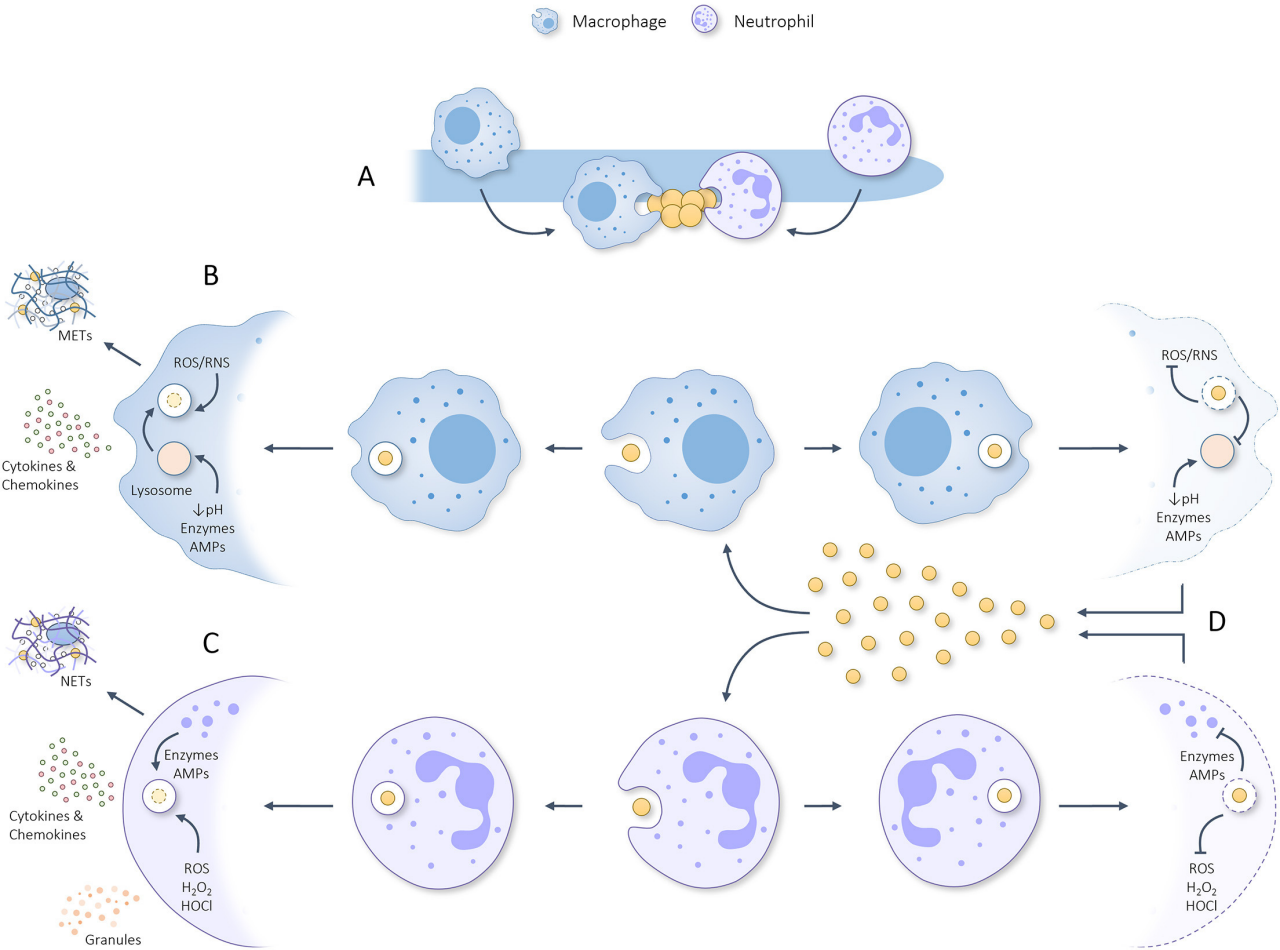
Graphical summary regarding **(A)** the attraction of macrophages and neutrophils toward *C. albicans* hyphae which sequentially are scavenged for parts/attached microbes that can be phagocytosed. **(B)** Once phagocytosed by macrophages, the phagosome containing *S. aureus* will be fused with a lysosome to form a phagolysosome that utilizes ROS, RNS, acidity, degrading enzymes and AMPs to eliminate the phagocytosed threat. In response to phagocytosing *S. aureus*, macrophages can produce METs and secrete pro-inflammatory cytokines/chemokines which help attract/activate T cells, NK cells, dendritic cells and neutrophils. **(C)** Once phagocytosed by neutrophils, granules start fusing with the phagosome during a process deemed degranulation and utilize various kinds of ROS, degrading enzymes and AMPs to eliminate the phagocytosed threat. In response to phagocytosis/activation, neutrophils can produce NETs and secrete granules plus pro-inflammatory cytokines/chemokines. **(D)** *S. aureus* has developed numerous ways to inhibit phagocytic killing by macrophages and neutrophils rendering it able to survive the harsh phagosomal/phagolysosomal environment, propagate, and kill the concerning phagocyte. Released staphylococcal cells can, thereafter, be phagocytosed to repeat the process. This misemployment of phagocytes could aid *S. aureus* dissemination to various other body sites and initiate lethal infection.

Oral polymorphonuclear leukocytes (PMNs), mainly neutrophils, constantly migrate into the oral cavity and patrol mucosal surfaces to detect and respond to present microorganisms [56, 57]. When PAMPs are detected, neutrophils phagocytose the pathogen (when possible) and release neutrophil extracellular traps (NETs), reactive oxygen species (ROS), cytokines, chemokines, and granules containing, amongst others, the serine proteases elastase, cathepsin G and proteinase 3 (Figures 1, 2) [27, 58–60].

Neutrophils are attracted to the site of infection by locally secreted cytokines and chemokines where they also directly respond to candidal cell wall components via both their PRRs (TLR2, TLR4, TLR9, Dectin-1, Dectin-2, Dectin-3, DC-SIGN, and MINCLE) and secreted *C. albicans* Sap proteins [27, 61]. While phagocytosis of *C. albicans* yeast cells and smaller hyphae is still feasible, phagocytosis of larger hyphae present during candidiasis is not possible [62–64]. *C. albicans* cells that are phagocytosed can resist neutrophil killing to a certain extent but are, nevertheless, effectively blocked in their survival, growth, and escape [65, 66]. Regarding hyphae that cannot be phagocytosed, NETs majorly contributes to candidal killing. Evidently, *C. albicans* hyphae are more prone to trigger NET release compared to yeast cells [67]. NETs are networks of extracellular fibers primarily made up of neutrophil DNA and aid in hyphal killing by trapping *C. albicans* cells and binding/concentrating present antimicrobial factors [67, 68]. Oral PMNs release 13 times more NET material compared to circulating PMNs, rendering NETs particularly important in oral immunity [69]. Additional to phagocytosis and NETs, neutrophils are generally able to damage/kill fungal cells by releasing ROS which damage fungal and bacterial DNA, RNA and proteins [70]. However, neutrophilic killing mechanisms described above have been mainly studied using non-oral PMNs and planktonic cultures. *C. albicans* biofilms actually reduce the release of NETs and ROS, inhibiting the main antifungal effectors of neutrophils [71, 72]. Besides aiding in *C. albicans* killing, neutrophils also act as immune stimulators by releasing serine proteases such as elastase or cathepsin G [73]. These proteases act as immune stimulators by: (1) promoting pro-inflammatory cytokine and chemokine production [59, 74–76], (2) promoting secretion and aggregation of thrombocytes [77], (3) activating natural killer (NK) cells, T-cells and B lymphocytes, (4) increasing NK cell cytotoxicity and cytokine production [78], and (5) activating various chemoattractants [78–80].

In contrast to *C. albicans* hyphae, *S. aureus* cells can be readily phagocytosed by neutrophils. Following phagocytosis, phagosomes containing *S. aureus* utilize PMN-derived oxygen-dependent factors such as O^2−^, hydrogen peroxide (H_2_O_2_), hypochlorous acid (HOCl) and other ROS to kill the internalized threat (Figure 2) [81, 82]. Oral PMNs have been shown to be functionally capable of producing and utilizing ROS (both intracellular and extracellular) for microbial killing [57]. Additionally, neutrophils utilize PMN-derived oxygen–independent factors, including, elastase, proteinase-3, azurocidin, cathepsins, lysozyme, and various AMPs which are introduced into the phagosome by various granules during a process known as degranulation (Figure 2) [83–85]. Using various degranulation markers oral PMNs have been shown to have upregulated degranulation and increased exocytosis of granular content compared to peripheral PMNs [57]. The state of degranulation in oral PMNs has been suggested to be adjusted in accordance to the number of present oral PMNs relative to the bacterial load with the expression of degranulation markers increasing most during periodontitis [57]. Together, the oxygen-dependent and independent factors elicit highly effective antibacterial effects. Even though the studies mentioned above mainly focus on general PMN responses, similar responses are likely to occur inside the oral cavity. Interestingly, even though oral PMNs are more prone to adhere and internalize bacteria compared to circulating PMNs, they show a reduced capacity for *E. coli* and, therefore, possibly *S. aureus* as well [69]. The reduced killing efficacy of PMNs is limited further by the ability of *S. aureus* to inhibit phagocytosis and phagosomal killing [46, 47]. Importantly, *S. aureus* is able to prevent oxidative phagosomal killing by; (1) utilizing two superoxide dismutases to convert superoxide into hydrogen peroxide and molecular oxygen, (2) degrading hydrogen peroxide to water and oxygen, (3) producing the antioxidant carotenoid staphyloxanthin, (4) repairing oxidative damaged proteins, and (5) by directly decreasing ROS production [46, 47]. Moreover, AMPs utilized during non-oxidative killing can be impaired by *S. aureus*, as discussed earlier, through cell surface alterations and secretion of AMP inhibiting/degrading proteins. By inhibiting neutrophilic oxidative and non-oxidative killing mechanisms *S. aureus* cells can survive inside the phagosome, propagate, and produce cytolytic toxins which induce neutrophil osmotic lysis and necrosis [46, 47]. Subsequentially, viable *S. aureus* cells are released into the surrounding environment following which the process of phagocytosis, survival, staphylococcal propagation and neutrophil death can be repeated (Figure 2). If this process occurs outside the original invasion zone, *S. aureus* cells could be able to utilize this mechanism as means for dissemination. Additional to phagocytosis, neutrophils secrete NETs, ROS, pro-inflammatory cytokines and chemokines in response to *S. aureus* [46, 47, 86]. However, *S. aureus* is a potent neutralizer of NETs by using nuclease and adenosine synthase to convert them to deoxyadenosine [46, 87, 88]. Staphylococcal NET neutralization could provide *C. albicans* with protection against this anti-hyphal immune response. Besides phagocytosis and NET circumvention, *S. aureus* is able to inhibit neutrophil chemoattraction, PRR activation, extravasation, calcium mobilization, and actin polymerization [47]. Nevertheless, during co-infection with *C. albicans*, phagocytes are still actively attracted to the hyphae regardless of *S. aureus*, suggesting the chemotactic inhibition of *S. aureus* to either be suppressed during pathogenic co-culture or overruled by hyphal chemoattraction [11]. Finally, neutrophil secreted serine proteases elastase, proteinase 3, and cathepsin G are also inhibited by *S. aureus* [46, 47]. Therefore, *S. aureus* is indeed recognized and phagocytosed by neutrophils but has the ability to circumvent phagocytosis, survive within the phagolysosomes, and kill the neutrophil. Moreover *S. aureus* is able to reduce extracellular neutrophil responses that also affect *C. albicans*, granting both organisms protection during co-infection.

### The Complement System

The complement system is one of the first extra-epithelial antimicrobial immune responses and is actively present in human saliva and GCF [89–92]. Following mucosal damage or gingival inflammation, the complement system initiates fast and efficiently via a combination of the classical, alternative and lectin pathways plus indirectly via the coagulation and fibrinolysis systems [89]. Regarding *C. albicans* and *S. aureus* immunity the complement factors B, C3, C4, C5-C9, and mannan binding lectin (MBL) are considered the main effectors [89, 93, 94]. C5-C9 are known to form the membrane attack complex (MAC) which is characterized as the direct antifungal response of the complement system by forming pores in the cell surface [89]. Even though studies regarding complement responses to *C. albicans* and *S. aureus* have not yet been performed in relation to the oral cavity, the oral response is expected to act similarly.

However, even though MACs are considered antifungal, they are not able to effectively lyse candidal cells, indicating the complement system to play a more modulatory role in *C. albicans* immunity [89]. Immune modulation by the complement system is facilitated through opsonization of *C. albicans* cells with C3 and Mannose-binding lectin (MBL) which promote phagocytosis by local phagocytes when possible [95, 96]. Furthermore, MBL is able to stimulate ROS production by nearby neutrophils which are readily attracted to the site of infection via complement factor C5a, a cleavage product of C5 [97, 98]. However, *C. albicans* is able to evade the complement system by masking itself for complement activation, cleaving/blocking complement proteins and recruiting complement regulators [28, 89]. While the antifungal mechanisms governed by the complement system remain modulatory, its direct antibacterial effects are more prominent.

Regarding *S. aureus*, MAC formation is able to directly damage and kill the bacterium by successfully puncturing the bacterial cell surface [90]. The complement system is also capable to reduce staphylococcal/EC adherence [99]. However, this is likely overcome during pathogenic candidal growth since the presence of *C. albicans* is known to significantly enhance the presence of *S. aureus* on the tongue of immunocompromised mice [10, 11, 13]. Similar to *C. albicans, S. aureus* has developed various mechanisms to avoid complement mediated killing, including the inhibition of MAC formation [100], inhibition of the classical and lectin pathways [101], and the utilization of various extracellular proteases which efficiently degrade crucial complement factors [102]. Since both organisms possess mechanisms to inhibit the complement system, their inhibitory potency likely adds up during co-infection.

To summarize, antimicrobial proteins, AMPs, neutrophils, and complement factors present in saliva and GCF are able to balance microbial growth to prevent overgrowth and pathogenesis (Figure 1). Thereby, saliva and GCF play key roles in maintaining homeostasis in the oral cavity. However, numerous evasion strategies allow *C. albicans* and *S. aureus* to reduce antimicrobial efficacy and result in disease. Loss of immune fitness obviously increases the incidence of infections originating from the oral cavity. When pathogenic hyphal growth of *C. albicans* is not limited, hyphae will grow invasively into the oral epithelium and trigger intra-epithelial immunity. During invasive hyphal growth *S. aureus* will translocate into the oral tissue and contribute to the immune activation as well.

## Intra-Epithelial Oral Immunity

Once invaded, *C. albicans* and *S. aureus* stimulate intra-epithelial immunity, including the type 17 response, further attraction/activation of both neutrophils and macrophages (assisted by Th1 activation), the NK response, and B cell response (Figure 1). Moreover, intra-epithelial immunity promotes extra-epithelial immunity by stimulating the production/secretion of AMPs by salivary glands and epithelial cells.

### Type 17 Response

The type 17 response is mainly governed by antigen presenting cells, such as dendritic cells (DCs) and Langerhans cells (specialized subset of DCs), which patrol the soft oral tissues for foreign antigens [103]. Oral mucosa, except for sublingual mucosa, are relatively non-absorptive, indicating the need for antigens to penetrate the epithelium in order to induce downstream immune responses [104]. When DCs encounter fungal and/or bacterial antigens they are activated and start sampling the antigen and present it on the cell surface using major histocompatibility complex (MHC) class II molecules [105]. Following antigen uptake, DCs also start to mature and travel to the draining lymph nodes where naïve T-cells are able to bind DCs and their presented antigens. Following maturation, DCs produce different cytokines according to the PRR and molecular activation. Fungal and bacterial activated DCs produce, amongst others, IL-6 and TGFβ which, together with MHCII antigen binding and the supporting production of IL-1β and IL-23, drive naïve T-cells into Th17 differentiation (Figure 1) [105]. The main goal of differentiated Th17 cells is to migrate to infected tissues and support local immune responses by producing IL-17, IL-22 and IFN-γ. Additional IL-17 is produced by local cells of the innate immune system, including γδ T cells, mucosa-associated invariant T (MAIT) cells, NK T cells, innate lymphoid cells (ILCs), T cell receptor αβ^+^ cells (TCR αβ^+^), natural Th17 cells (nTh17) and Foxp3^+^ T regulatory cell (Treg)–like cells which, together with Th17 cells, are deemed the type 17 response [106, 107]. Even though cells of the type 17 response only constitute a small fraction of the oral cell population (i.e. 1% of the gingival CD4 T cell population) they are indispensable for intra-epithelial immunity [27, 103]. Additional to intra-epithelial immunity, the type 17 response stimulates extra-epithelial oral immunity through IL-17 induced production/secretion of AMPs and chemokines [27, 103]. Th17 produced IL-22 plays an intricate role in epithelial repair by stimulating survival and proliferation of the basal oral epithelium to replenish damaged IL-17 receptor-expressing ECs [108]. Moreover, IL-22 is responsible for preventing accumulation of apoptotic inflammatory cells and preservation of genome integrity [108]. In summary, even though the type 17 response does not directly kill fungi or bacteria, it does crucially stimulates other aspects of oral antifungal/bacterial immunity.

*C. albicans* is able to activate the type 17 response via hyphal PAMPs and through candidalysin. Candidalysin activates the type 17 response by activating oral ECs, as discussed earlier, into releasing IL-1α/β which stimulate the proliferation of innate Th17 lymphocytes. Moreover, candidalysin synergistically acts with IL-17 to promote more oral EC activation and, thus, creating a positive feedback loop of candidalysin induced type 17 activation [109]. However, *C. albicans* has developed mechanisms to sense IL-17 and induce autophagy, resulting in efficient recycling of nutrients and cellular components, clearance of aggregates and improved resistance to environmental stresses such as antifungal drugs [110]. The binding of IL-17 also increases candidal adhesion and filamentous growth resulting in the formation of biofilms which are known to be more resistant to antifungal immune responses and treatments [110, 111].

Whereas *C. albicans* is able to use the type 17 response to induce autophagy for emergency survival, *S. aureus* is able to reduce T-cell numbers by producing various toxins that directly kill local T-cells [112]. Moreover, staphylococcal cell wall components and produced phenol soluble modulins have been found to suppress T cell proliferation and differentiation [112, 113]. *S. aureus* is also able to stimulate the expansion of T-cell suppressive immune cells such as granulocytic and monocytic myeloid-derived suppressor cells [112, 113]. Together, IL-17 triggered biofilm formation of *C. albicans* could grant *S. aureus* protection while generic T cell inhibition of *S. aureus* further reduces type 17 response efficacy during co-infection.

### Macrophages and Neutrophils

In contrast to neutrophils, macrophages are not present outside of the oral epithelium and, therefore, only contribute to intra-epithelial immunity. Oral macrophages react to invaded microbial threats by phagocytosing and killing them (Figures 1, 2). Similar to neutrophils, macrophages cannot phagocytose larger intact hyphae but scavenge hyphal cells for associated microbes that can be phagocytosed [11, 62–64].

The attraction to and recognition of *C. albicans* cells by macrophages is stimulated by fungal cell wall glycosylation [63, 64] and involves both PRRs (TLR2, TLR4, TLR9, Dectin-1, Dectin-2, Dectin-3, DC-SIGN, and MINCLE) and recognition of secreted *C. albicans* Sap proteins [27, 61]. Following *C. albicans* recognition and/or phagocytosis, macrophages secrete pro-inflammatory cytokines and chemokines [27]. Together with the cytokines and chemokines secreted by oral ECs, these molecules help attract and activate more macrophages and neutrophils [27]. Macrophages can contribute to *C. albicans* killing through phagocytosis and producing macrophage extracellular traps (METs) [114]. Following phagocytosis *C. albicans* is, however, able to alter its transcriptional profile to survive nutrient-depleted and acidic phagosomes [115]. Furthermore, *C. albicans* induces cell wall remodeling to initiate an intraphagocytic yeast to hyphal transition able to induce pyroptosis, an inflammatory mediated lytic programmed cell death, as well as rupturing of the macrophage membrane [115]. METs contribute to the antifungal response similar to NETs and are likely released before *C. albicans* is able to induce macrophage pyroptosis [116]. As discussed for extra-epithelial immunity, neutrophils prevent overgrowth of *C. albicans* yeast cells by phagocytosis, prevent/limit hyphal growth by releasing NETs and ROS, and stimulate further immune activation by secreting serine proteases.

In line with their general response, macrophages play a crucial role in the immune response against *S. aureus* infections (Figure 2) [49]. Macrophages recognize *S. aureus* through a combination of receptors, including scavenger receptors, complement receptors and Fc receptors [117]. *S. aureus* activated macrophages produce various cytokines and chemokines which attract T cells, NK cells, DCs, more macrophages, and neutrophils to the site of infection (Figures 1, 2) [118]. When activated, macrophages engulf staphylococcal cells into a phagosome which, unlike in neutrophils, fuses with a lysosome to form a bactericidal phagolysosome (Figure 2) [117]. Following *S. aureus* interaction, macrophages can either provoke a pro-inflammatory or an anti-inflammatory response. The pro-inflammatory response entails active phagocytosis, the production of intracellular ROS, nitric oxide (NO), pro-inflammatory cytokines, AMPs (including hepcidin and calprotectin), and enzymes, as well as acidification of the phagolysosome, nutrient restriction, and autophagy [49]. Similar to the antifungal response, macrophages target extracellular bacteria using macrophage extracellular traps (METs) [114]. In response to recurrent *S. aureus* infections macrophages are able to generate a longer term memory by increasing their pro-inflammatory responsiveness [119]. Importantly, recurrent *S. aureus* infections also induce a Th1 memory response which results in additional IFN-γ production and macrophage activation [120–122]. In contrast, *S. aureus* infections involving mechanisms such as biofilm growth induce an anti-inflammatory macrophage response, actually impairing phagocytosis [123]. Additionally, *S. aureus* is able to avoid macrophage killing by preventing phagolysosome acidification [124], restricting ROS/RNS production [125], and preventing autophagy by blocking autophagic flux [126]. Using these mechanisms, *S. aureus* is able to survive inside macrophages, proliferate and ultimately kill the phagocyte, resulting in the release of viable *S. aureus* cells which are again internalized by other macrophages to repeat the process (Figure 2) [124, 127, 128]. When co-phagocytosis of *C. albicans* and *S. aureus* occurs, the survival mechanisms utilized by both organisms could enhance intracellular survival and macrophage killing.

Even though the intra-epithelial neutrophil response operates identically to the extra-epithelial response discussed earlier, there are some additions. Namely, intra-epithelial neutrophils are able to interact with, activate and act as antigen presenting cells (APCs) [129]. Moreover, produced NETs activate APCs and directly prime T cells, highlighting the importance of neutrophils in both antifungal immunity and immune regulation [129]. Due to the fact that the intra-epithelial macrophage and neutrophil responses are stimulated by all aspects of *C. albicans*/*S. aureus* oral immunity (Figures 1, 2) they can be considered the main effectors.

### Natural Killer Cells

Besides the type 17, macrophage, and neutrophil response, NK cells also contribute to intra-epithelial *C. albicans*/*S. aureus* immunity. Whereas NK cells constitute approximately 5–20% of the peripheral blood mononuclear cell population, they comprise a substantial part of the gingival lymphoid cell population and elicit both direct and indirect antifungal responses by phagocytosis and releasing cytotoxic granular content [130, 131].

Direct antifungal responses mainly involve phagocytosis and the release of granular contents, including the apoptosis such as granzyme B and the pore-forming molecules granulysin and perforin [130, 132, 133]. Due to the fact that depletion of NK cells in immunocompetent mice does not increase *C. albicans* susceptibility, NK cells are expected to play a more immunomodulatory role in *C. albicans* immunity [133]. Immune modulation by NK cells is achieved through production of inflammatory mediators able to attract and activate macrophages and neutrophils [130, 132, 133]. NK cell cytokine production is also induced by activated DCs and contributes to sustaining neutrophil antifungal response [134]. While most NK cell studies have mainly been focussed on systemic fungal infections, similar NK cell functions are likely to contribute to mucosal *C. albicans* immunity [27].

Regarding antibacterial responses, NK cells elicit both direct and indirect effects [135]. Direct antibacterial effects are facilitated by inducing microbial apoptosis through releasing granular components and various AMPs [135, 136]. Indirect NK cell responses again mainly include the production of inflammatory mediators. NK cells, while mainly activated by type 17 cytokines, are also able to be activated directly by *S. aureus* and by staphylococcal products, likely contributing to the control of staphylococcal infections prior to T cell activation [137–140]. Interestingly, even though depletion of NK cells does not influence *C. albicans* susceptibility, it does increase the susceptibility to staphylococcal infections [139]. This is partly attributable to an NK cell induced promotion of *S. aureus* phagocytosis by macrophages [139]. Research regarding evasion strategies of *C. albicans* and *S. aureus* against the NK cell response is, unfortunately, still lacking.

### B Cells

Finally, the B cell response also contributes to intra-epithelial oral immunity, albeit playing a minor role in *C. albicans*/*S. aureus* immunity [141–144].

While patients suffering from agammaglobulinemia hardly or do not produce immunoglobulins they do show a normal functioning antifungal immune response [144]. Nevertheless, patients undergoing B cell depletion therapy exhibit an increased susceptibility to fungal infections attributable to reduced Th1/Th17 responses, whereas bacterial infection rates remain relatively normal, indicating B cells to contribute to *C. albicans* immunity in an immunoglobulin independent manner [141, 142].

In contrast to antifungal immunity, B-cell-deficient mice do not show a difference in bacterial infection related mortality and clearance, indicating B cells to not significantly contribute to *S. aureus* immunity [143]. Nevertheless, vaccination of mice with a sublethal doses of live *S. aureus* does provoke the production of specific antibodies against a wide variety of staphylococcal antigens [145]. Correspondingly, patients suffering from *S. aureus* skin and soft tissue infections, prosthetic joint infections, and pediatric hematogenous osteomyelitis establish a repository of memory B cells which are able to produce antibodies against a variety of *S. aureus* exotoxins [146, 147]. However, immunoglobulins acting directly on *S. aureus* have not yet been identified, which is likely attributable to the fact that B cells are not able to directly bind *S. aureus* cells [148].

Altogether, the above mentioned extra-epithelial immune responses inhibit propagation of invaded *C. albicans* and *S. aureus* while, additionally, stimulating the repair of damaged tissue. Even though *C. albicans* and *S. aureus* have developed numerous mechanisms to evade these immune responses, the oral immune system of healthy individuals is capable of preventing and/or limiting their pathogenicity to maintain homeostasis. However, in immunocompromised hosts this no longer holds true, allowing for pathogenic growth of *C. albicans* and with it possibly the initiation of *S. aureus* BSIs.

## Immune Dysfunction Facilitates Opc Induced *S. aureus* BSIs

To date, *in vitro* and murine model studies regarding oral polymicrobial *C. albicans/S. aureus* infections have established that; (1) immune suppression is essential for the development of OPC, (2) OPC increases the presence of *S. aureus* on the tongue surface, (3) invasive OPC facilitates infiltration of *S. aureus* into the underlying substratum via, amongst others, the production of candidalysin, (4) severe immune suppression significantly reduces dissemination of *S. aureus* [10–13, 15]. Therefore, the role of the oral immune system appears to be paradoxical: whereas low levels of suppression allow for OPC development and *S. aureus* dissemination, severe suppression significantly reduces the dissemination potency of *S. aureus*, even though OPC development is more severe. The immune system in immunocompromised individuals could, thereby, first allow invasive candidal growth due to lack of activity while remaining immune activity sequentially aids in *S. aureus* dissemination. However, while immune deficiencies, either congenital (primary) or acquired (secondary), are known predisposing factors for OPC and *S. aureus* BSIs separately, they have not yet been described with regard to OPC induced staphylococcal BSIs [149–152]. Therefore, predominant immune deficiencies separately known to increase the risk for OPC as well as *S. aureus* BSIs will be discussed.

The vast majority of immune deficiencies related mucocutaneous candidiasis incidences are attributable to diabetes mellitus, cancer, human immunodeficiency virus (HIV) infection, and prescribed corticoids [152–157]. *S. aureus* BSIs have been associated with similar immune deficiencies, fortifying the notion that immune deficiency induced OPC could concordantly increase the risk of *S. aureus* BSI development [149–151, 158]. Interestingly, once instigated, *S. aureus* BSIs do not show a higher mortality rate among immunocompromised patients, indicating immune deficiencies to only contribute to BSI onset but not further lethality [159, 160]. Due to the fact that diabetes mellitus, HIV, cancer, and use of prescribed corticoids comprise the majority of immune deficiencies linked to both *C. albicans* and *S. aureus* infections separately, their effects on the oral immune response to both organisms will be discussed. Additional immune deficiencies, affecting aspects of the oral *C. albicans* and *S. aureus* immune response, are listed in Table 1.

**Table 1:** Primary, secondary, and drug induced immune deficiencies able to affect the various aspects or oral *C. albicans* and *S. aureus* immunity.

**Immune component**	**Primary deficiencies**	**Secondary deficiencies**	**Drug induced deficiencies**	**References**
Complement system	Deficiencies in: C1-C9, mannan-binding lectin, MASP-2, factor B, factor D, factor H, factor I, CR1, and CR3,	Diabetes, malnutrition	Complement inhibitors	[161–165]
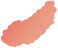 Salivary glands (Saliva)	Ectodermal dysplasia, cystic fibrosis, and Prader-Willi syndrome	Cancer, ionizing radiation, Sjögren's syndrome, systemic lupus erythematosus, mixed connective tissue disease, sarcoidosis, amyloidosis, Crohn's disease, ulcerative colitis, diabetes, hyper-and hypothyroidism, Cushing syndrome, Addison disease, depression, narcolepsy, Parkinson's disease, Bell palsy, Alzheimer's disease, Holmes-Adie syndrome, eating disorders, anorexia nervosa, bulimia, anemia, atrophic gastritis, dehydration, alcohol abuse, HIV/AIDS, epidemic parotitis, Epstein-Barr virus, bacterial sialadenitis, tuberculosis, hypertension, fibromyalgia, chronic fatigue syndrome, burning mouth syndrome, compromised masticatory performance, surgery, trauma, gland stones, and sialadenitis	Cannabis, ecstasy, various: antidepressants, alpha-receptor antagonists, antipsychotics, antihistamines, diuretics, antihypertensive agents, appetite suppressants, decongestants, bronchodilators, skeletal muscle relaxants, antimigraine agents, opioids/hypnotics, H2 antagonists/proton pump inhibitors, cytotoxic drugs and anti-HIV drugs, muscarinic receptor antagonists, alpha receptor antagonists, beta blockers, ACE inhibitors, atropinics, benzodiazepines, retinoids, radioiodine, and protease inhibitors	[166–170]
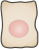 Epithelial cells		Cancer, ionizing radiation, surgery, trauma, oral lesions induced by, bacterial, fungal and viral infections or by associated dermatological diseases, recurrent aphthous stomatitis, inflammatory bowel diseases, and nutritional deficiencies in B12 and folate	Antimalarials, gold salts, Non-steroidal anti-inflammatory drugs, ACE inhibitors, HIV protease inhibitors, antihypertensive agents, phenothiazines, sulphonamides, tetracyclines, thiazide diuretics, mTOR inhibitors, chemotherapy agents, mycophenolate mofetil, thiol radical–containing drugs, antipsychotic medications, spironolactones, sulphonamides, infliximab, adalimumab, antimicrobials, anticonvulsants, calcium channel blockers, calcineurin inhibitors, and phenytoin	[171–180]
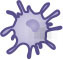 Dendritic cells	IRF8 & GATA2 deficiencies, reticular dysgenesis, WHIM syndrome, bare lymphocyte syndrome, Wiskott-Aldrich syndrome, CD40/CD40L deficiency, Pitt-Hopkins Syndrome, hyper-IgE syndrome, and IRF7 mutations	Ionizing radiation	Aspirin, deoxyspergualin, mycophenolate mofetil, N-Acetyl-l-cysteine, vitamin D3 analogs, antiproliferative agents, corticoid steroids, Janus kinase inhibitors, calcineurin inhibitors and mTOR inhibitors	[181–183]
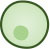 Type 17 cells	Autosomal dominant hyper-IgE syndrome, STAT-1 mutations, auto-immune poly endocrine syndrome type 1, hyper-IgM syndrome, chronic mucocutaneous candidiasis, deficiencies in IL-17R, IL-17F, IFN-γ and IL-12, DiGeorge syndrome, Ataxia telangiectasia, Wiskott-Aldrich syndrome, X-linked lymphoproliferative syndrome, MHC deficiency, and Cartilage-hair hypoplasia	Diabetes, HIV, cancer, aging, hypoproteinaemia, diabetes mellitus, UV-light exposure, viral infections involving the measles virus, cytomegalovirus, and influenza virus	Corticoid steroids, Janus kinase inhibitors, calcineurin inhibitors, TNF-α inhibitors, IL-1 inhibitors, IL-6 inhibitors, IL-17 inhibitors, cytotoxic agents, panlymphocyte depleting agents, mTOR inhibitors, and antimetabolites	[164, 173, 184–190]
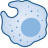 Macrophages	Chronic granulomatous disease, Chédiak–Higashi syndrome, IL12/IFN-γ defects, cystic fibrosis, Niemann–Pick disease, Gaucher disease, Krabbe's disease, metachromatic leukodystrophy, and Fabry's disease	Diabetes, cancer, Whipple's disease, atherosclerosis, and malnutrition	Corticoid steroids, Janus kinase inhibitors, calcineurin inhibitors, polyclonal antithymocyte Globulins, and mTOR inhibitors	[173, 188, 191–200]
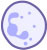 Neutrophils	Severe congenital neutropenia, cyclic neutropenia, Shwachman-Diamond syndrome, Chédiak-Higashi syndrome, leukocyte adhesion deficiency, type 2 Griscelli syndrome, chronic granulomatous disease, Mendelian susceptibility to mycobacterial disease, type 2 Hermansky-Pudlak syndrome, p14 deficiency, WHIM syndrome, CD40 ligand deficiency, Agammaglobulinemia with absent B-cells, purine nucleoside phosphorylase deficiency, autoimmune lymphoproliferative syndrome, cartilage hair hypoplasia, glycogen storage disease Ib, Barth syndrome, dyskeratosis congenita, reticular dysgenesis, Cohen syndrome, Niemann–Pick disease, Gaucher disease, Krabbe's disease, metachromatic leukodystrophy, and Fabry's disease	Diabetes, HIV, large granular lymphocytic leukemia, Protein-calorie malnutrition, folate/vitamin B12 shortage, and chemotherapy	Phenothiazines, antithyroid medications, corticoid steroids, Janus kinase inhibitors, calcineurin inhibitors, polyclonal antithymocyte globulins and chloramphenicol	[173, 195, 201–204]
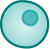 NK cells	Absolute, classical and functional NK cell deficiency, xeroderma pigmentosum, Bloom's syndrome, ataxia telangiectasia, Fanconi's anemia, bare lymphocyte syndrome, familial erythrophagocytic lymphohistiocytosis, Chediak–Higashi syndrome, Griscelli syndrome, Papillon-Lefevre, Hermansky-Pudlak, X-linked lymphoproliferative syndrome, leukocyte adhesion deficiency, X-linked hyper-IgM syndrome, paroxysmal nocturnal haemoglobinuria, von Hippel–Lindau, autoimmune lymphoproliferative syndrome, Wiskott–Aldrich syndrome, IL-12 receptor deficiency, X-linked agammaglobulinemia, NF-kB essential modulator deficiency, ectodermal dysplasia with immunodeficiency, common variable immunodeficiency, and chronic mucocutaneous candidiasis	Diabetes, chronic fatigue syndrome, obesity, and high-dose ionizing radiation	Corticoid steroids, Janus Kinase inhibitors, calcineurin inhibitors, IL-17 inhibitors, and mTOR inhibitors	[163, 188, 205–210]

### Diabetes Mellitus

The global burden of diabetes is increasing continuously and is currently responsible for over 1.5 million deaths per year, ranking it within the top 10 causes of death [211, 212]. Individuals suffering from diabetes are more susceptible to *C. albicans* and *S. aureus* infections due to both a reduced salivary flow and impaired immune responses, including suppression of cytokine production, defects in phagocytosis, dysfunction of immune cells and a decreased complement response [213–215]. Evidently, peripheral blood mononuclear cell (PBMCs) isolated from diabetic patients have a lowered cytokine production and take on a more anti-inflammatory phenotype [216–218]. However, macrophages do increase their phagocytosis rate in response to *C. albicans* or *S. aureus* during hyperglycaemia [216, 217]. Therefore, even though macrophages increase bacterial phagocytosis, they have a reduced intracellular killing efficacy and lowered immune stimulatory capacity [218]. Besides macrophages, neutrophils of diabetic patients are impaired in their attraction, migration, ROS production, NET formation, and degranulation [213]. Hyperglycaemia is also known to decrease the efficacy of NK cells and the complement system by inhibiting their degranulation capacity and C3/C4 pathway activation, respectively [213, 218]. Interestingly, under hyperglycaemic conditions *S. aureus* is able to deplete serum C3 levels, reducing complement-mediated killing as well [219]. Altogether, hyperglycaemia allows for invasive *C. albicans* and *S. aureus* growth due to a reduced salivary flow and impaired extra-epithelial neutrophil and complement responses. Once invaded, the reduced intra-epithelial type 17, macrophage, neutrophil, and NK cell responses could allow for sequential phagocytic uptake, survival and dissemination of *S. aureus*. Interestingly, the risk of developing diabetes is significantly higher amongst individuals suffering from HIV infections [220].

### HIV

Approximately 37.7 million individuals live with HIV/AIDS worldwide while HIV-related diseases are responsible for about 680,000 deaths each year [221]. HIV especially burdens low-income countries, ranking it within the top 10 of causes of death [222]. One of the main causes of HIV induced mortality is a secondary infection which develops due to a progressive decline of CD4 T cells [223]. The predominantly affected CD4 T cell class during HIV infections is Th17, resulting in both cellular depletion and dysfunction [224–226]. Four mechanisms are known to inflict HIV induced T cell decline; (1) direct viral killing of infected cells, (2) induced apoptosis of infected cells, (3) killing of infected cells by CD8 T cells, and (4) defects in T cell regeneration together with destruction of CD4 T cell progenitors within the thymus [223]. The decline in CD4 T cells will eventually reach a critical point at which the immune system is no longer capable of preventing opportunistic pathogenic infections of, for instance, *C. albicans* and *S. aureus*. This is well reflected by the fact that oral candidiasis induced lesions are commonly observed during HIV infections and are significantly associated with a low CD4 T cell count [227–231]. Correspondingly, HIV patients are at an increased risk of developing *S. aureus* infections and BSIs which, besides T cell depletion, are also linked to reduced neutrophil counts (neutropenia) [232–235]. Neutropenia is frequently observed among HIV patients and is strongly associated with reduced CD4 T cell numbers and an increased viral load [236]. Importantly, neutrophils still present during HIV infections are known to actually contribute to T cell dysfunction by suppressing T cell responses [237]. Besides T cells, macrophages and dendritic cells can also be infected with HIV. However, macrophages are known to resist HIV infection much better than CD4 T cells besides which they are less sensitive to cytotoxic killing of the virus, also producing and releasing relatively low amounts of viral particles when killing does occur [238]. Taken together, the reduction in extra-epithelial neutrophils most likely allows for invasive OPC development which is able to progress due to intra-epithelial CD4 T cell depletion, also reducing extra-epithelial AMP responses and barrier repair. Following the development of invasive OPC, *S. aureus* will co-invade and likely utilize the reduced intra-epithelial neutrophil count to disseminate. Interestingly, the immunocompromised state of HIV patients also significantly increases their risk of developing various types of cancer linked to *C. albicans* and *S. aureus* infections [239, 240].

### Cancer

Cancer is currently responsible for 10 million deaths per year, ranking it as one of the leading causes of mortality [241]. Besides its direct lethal effects, cancer and its treatments also increase the risk for infection [242, 243]. The increased risk of infection observed among cancer patients depends on various risk factors such as the type of cancer (i.e. lymphoma or acute leukemia versus a solid tumor), reduced cellular functioning as a result of cytotoxic or immunosuppressive therapies, usage of indwelling devices (e.g. catheters), the severity/duration of induced neutropenia, surgery, and gastrointestinal mucositis due to chemotherapy [243]. Due to the vast variation in cancer types and treatments it is not possible to summarize their effects on the aspects of oral immune responses relevant to *C. albicans* and *S. aureus* infections. Nevertheless, studies concerning human cancers and murine cancer models and have shown various relevant cancer induced peripheral immune perturbations which have been extensively reviewed elsewhere [244]. It must be noted that not every perturbation is yet known to occur in every cancer type. Known perturbation related cancer types and mouse models are summarized in detail by Hiam-Galvez et al. [244]. In brief, cancer is able to induce: (1) expansion of immature immunosuppressive neutrophils and monocytes due to aberrant haematopoiesis, (2) a decrease in dendritic cell subsets and dendritic cell maturation, (3) lymphopenia, (4) reduction of CD4 T cell function, (5) an increase in regulatory T and B cells able to dampen immune responses, and (6) a suppressed NK phenotype through a decreased number of activating receptors and cytotoxic potential as well as increased number of inhibitory receptor numbers [244]. Interestingly, surgical resection of the tumor can reverse various of these peripheral immune perturbations in multiple murine breast and colon cancer models, suggesting the observed immune effects to indeed be induced by the tumor itself [245]. Wheter this also holds true for humans and other cancer types remains to be determined. Besides the cancer itself, treatments can also affect peripheral immune responses but remain highly dependent on the type of treatment and cancer [244]. Treatment induced perturbations, include neutropenia, lymphodepletion, myeloid cell expansion, reduced APC function and T cell responses, and increased numbers of immunosuppressive polymorphonucleocytes and monocytes [244, 246, 247]. Additionally, various chemotherapies and radiation therapy affecting the oral cavity can inflict damage to the epithelial lining of the oral cavity and the salivary glands, allowing oral infections to instigate more easily [171, 172, 247–249]. Moreover, high dosage radiation therapies covering the oral cavity can eliminate small oral mucosal blood vessels and/or result in severe mucositis with ulceration which both induce necrosis of oral soft tissues [243]. Radiation may also damage the serous cells of salivary glands, leading to xerostomia (dry mouth) which, in relation to neck and head radiation, is significantly linked to an increased risks of *C. albicans* and *S. aureus* infections [248, 249].

Altogether, no general claim can be made concerning cancer and treatment induced peripheral immune perturbations. However, it remains clear that induced immunosuppression, damage the oral epithelial barrier, and induced xerostomia can allow for OPC development and more easy invasion of *C. albicans* and *S. aureus*. Interestingly, corticoids, the remaining risk factor for *C. albicans* and *S. aureus* infections, are commonly administered to cancer patients for their anti-cancer and anti-swelling effects [250].

### Corticoids

Corticoids, while not considered as direct immunocompromised conditions, are commonly prescribed immunosuppressants and are utilized to induce immunosuppression in murine co-infection models involving *C. albicans* and *S. aureus* [12–14, 251]. Whereas the positive feedback loop of the *C. albicans*/*S. aureus* immune response triggers pro-inflammatory effects (Figure 1), endogenous glucocorticoids play an essential role in both limiting and resolving inflammation to prevent adverse effects [252]. Corticoids are derived from the glucocorticoid family of steroid hormones and are, therefore, able to dampen immune responses in inflammatory disorders such as allergies, asthma, and autoimmune diseases, besides which they are beneficial for other disease such as cancer [173, 223, 250]. Importantly, corticoids are also generally used in murine models to induce an immunocompromised state. The immunosuppressive effects of corticoids are mainly facilitated through binding intracellular glucocorticoid receptors expressed by the majority of human cell types [223, 253]. Due to the fact that multiple cell types express these receptors, corticoids induce multiple anti-inflammatory effects. It is generally accepted that corticoids reduce the level and function of CD4 T cells besides which they reduce pro-inflammatory responses of macrophages and monocytes, resulting in a reduction of: (1) pro-inflammatory cytokine production, (2) chemotaxis, (3) migration, (4) phagocytosis, (5) oxidative burst and free radical generation, and (6) nitric oxide secretion [223, 253–256]. However, various studies regarding corticoid treatment and neutrophils have also reported upregulation of pro-inflammatory responses besides which there have been contradictory studies regarding the inhibition/stimulation of corticoid induced neutrophil apoptosis, highlighting the complexity of the corticoid response [256]. This complexity is further emphasized by Th17 glucocorticoid sensitivity. While Th17 cells were first considered insensitive to corticoids, Th17 cells in patients suffering from psoriasis and related disorders are corticoid sensitive, indicating corticoid sensitivity to also depend on the immunopathology [257]. Nonetheless, the anti-inflammatory effects of corticoids on lymphocytes, neutrophils, monocytes/macrophages, and other immune effector cells are known to increase the susceptibility to invasive candidiasis [254]. Additionally, prognosis of *S. aureus* bacteraemia is negatively affected by the use of corticoid steroids and other immunosuppressives [258–261]. Even though the relation between *C. albicans/S. aureus* infections and corticoid use is apparent, the mechanism driving this relation remains unclear.

To summarize, the most common immune deficiencies linked to both *C. albicans* infections and *S. aureus* BSIs include diabetes mellitus, HIV, cancer, and prescribed corticoids. Diabetes induces immune deficiency through xerostomia and reduced extra-epithelial neutrophil and complement responses as well as impaired intra-epithelial type 17, macrophage and neutrophil responses. The reduction of extra-epithelial neutrophils during HIV infections likely allows for OPC development which is able to progress due to reduced intra-epithelial Th17 responses. *S. aureus* could co-invade during OPC progression and utilize the reduction in neutrophil count to disseminate. Regarding cancer, immune deficiencies arise from various aspects of the disease and its treatment with induced neutropenia as the main effector. Finally, use of corticoids induces various immune deficiencies, including reduced type 17, neutrophil and macrophage responses. Each of these immune deficiencies are able to facilitate OPC development and could, thereby, account for *S. aureus* BSIs.

## Conclusion

A strict balance between pathogenic *C. albicans* growth and oral immune activation normally inhibits hyphal invasion and ensures oral homeostasis. When oral immunity is compromised OPC is able to develop and invade oral mucosal tissue. In murine models, when *S. aureus* is additionally present, it is able to co-invade with *C. albicans* and utilize phagocytes present in the tissue to disseminate to draining lymph nodes. Whether this holds true for human co-infections remains to be determined. Considering that: (1) *C. albicans* and *S. aureus* are a common member of the human oral microbiome [262], (2) approximately one in five cases of candidemia are polymicrobial [6], (3) *C. albicans* instigated *S. aureus* BSI, in theory, do not have to concur with candidemia, and (4) a significant number of patients suffering from staphylococcal BSIs have no reported porte d'entrée [9], human *S. aureus* BSIs could be facilitated by OPC. Immunocompromised individuals, additional to developing OPC, could, therefore, also be at risk of contracting concordant *S. aureus* BSIs. Considering the crucial contribution of the oral immune system in this process, the aim of this review was to provide a detailed overview concerning all relevant aspects of oral *C. albicans* and *S. aureus* immunity (Figure 1) and to discuss the predominant immune deficiencies able to allow for OPC induced *S. aureus* BSIs (Table 1). Based on current literature it is evident that when the oral epithelium is not broken due to physical intervention, loss of extra-epithelial oral immune fitness during immune deficiencies such as diabetes mellitus, HIV, cancer, and the use of corticoid steroids can facilitate invasive growth of *C. albicans* and result in breakage of the oral epithelial barrier. Through this porte d'entrée, oral bacteria associated with *C. albicans*, such as *S. aureus*, are granted access to the underlaying substratum. Sequentially, phagocytes are actively attracted to the site of invasion and start scavenging *C. albicans* hyphae while phagocytosing attached staphylococcal cells. *S. aureus* cells able to survive phagocytic killing can proliferate and kill the phagocyte, releasing more bacteria in the surrounding to repeat the process (Figure 2). In contrast to OPC development, this staphylococcal dissemination process is severely hampered by excessive immune suppression. Ultimately, *S. aureus* is either granted direct access to the bloodstream or by surviving inside phagocytes during migration. Once disseminated, *S. aureus* (either accompanied by *C. albicans* or not) can initiate lethal infections throughout the body. Due to the fact that various immune dysfunctions are able to instigate OPC, through loss of oral immune fitness, they are likely able to also function as facilitator of *S. aureus* BSIs and should, therefore, be considered a potent risk factor for OPC induced *S. aureus* dissemination. Besides *S. aureus, C. albicans* is able to interact with other oral BSI inducing bacteria such as *Staphylococcus epidermidis* [263]*, Enterococcus faecalis* [264], and *Streptococcus gordonii* [265, 266], the first, second, and fourth most common co-isolated bacteria during *C. albicans* BSIs, respectively [6]. Therefore, the described mechanism of oral *C. albicans* and immune system facilitated bacterial invasion and dissemination during immunosuppression could be a general BSI inducing mechanism for bacteria able to directly interact with *C. albicans*.

## Discussion

Even though candidemia has been found to coincide with bacterial BSIs in approximately 20% of the cases [6, 7], *C. albicans*-induced dissemination of bacteria has not yet been directly observed in humans. In contrast, studies using murine and *in vitro* models have provided strong evidence to support the risk of *C. albicans* induced *S. aureus* invasion and dissemination during immunosuppression [10–13, 15]. Moreover, while the overall structure of the immune system of mice and humans is similar, discrepancies do exist and have been extensively reviewed elsewhere [267]. Importantly, numerous *in vitro* studies used to investigate immune responses to *C. albicans* and *S. aureus*. While this response is often described as a general response, it could differ from *in vivo* oral immune responses. As OPC-facilitated invasion of *S. aureus* represents a new porte d'entrée with potentially life-threatening consequences, future research, unraveling the role of the oral immune system in, and the mechanism of, invasion and dissemination of bacteria such as *S. aureus* in humans are important next steps in development of life-saving preventive strategies.

## Author Contributions

RP wrote the original draft. BK, SZ, and SB reviewed and edited the manuscript and conceptualized the review. SB acquired funding. All authors contributed to the article and approved the submitted version.

## Funding

The work of RP in the laboratory of SB, in collaboration with the labs of BK and SZ, was funded by strategic investments of the University of Amsterdam in the framework of the Research Priority areas Host-Microbiome Interactions and Personal Microbiome Health.

## Conflict of Interest

The authors declare that the research was conducted in the absence of any commercial or financial relationships that could be construed as a potential conflictof interest.

## Publisher's Note

All claims expressed in this article are solely those of the authors and do not necessarily represent those of their affiliated organizations, or those of the publisher, the editors and the reviewers. Any product that may be evaluated in this article, or claim that may be made by its manufacturer, is not guaranteed or endorsed by the publisher.
